# New Motion Intention Acquisition Method of Lower Limb Rehabilitation Robot Based on Static Torque Sensors

**DOI:** 10.3390/s19153439

**Published:** 2019-08-06

**Authors:** Yongfei Feng, Hongbo Wang, Luige Vladareanu, Zheming Chen, Di Jin

**Affiliations:** 1 Faculty of Mechanical Engineering & Mechanics, Ningbo University, Ningbo 315211, China; 2 Parallel Robot and Mechatronic System Laboratory of Hebei Province and Key Laboratory of Advanced Forging & Stamping Technology and Science of Ministry of Education, Yanshan University, Qinhuangdao 066004, China; 3 Robotics and Mechatronics Department, Institute of Solid Mechanics of the Romanian Academy, 010141 Bucharest, Romania

**Keywords:** lower limb, rehabilitation robot, motion intention acquisition, static torque sensor

## Abstract

The rehabilitation robot is an application of robotic technology for people with limb disabilities. This paper investigates a new applicable and effective sitting/lying lower limb rehabilitation robot (the LLR-Ro). In order to improve the patient’s training initiative and accelerate the rehabilitation process, a new motion intention acquisition method based on static torque sensors is proposed. This motion intention acquisition method is established through the dynamics modeling of human–machine coordination, which is built on the basis of Lagrangian equations. Combined with the static torque sensors installed on the mechanism leg joint axis, the LLR-Ro can obtain the active force from the patient’s leg. Based on the variation of the patient’s active force and the kinematic functional relationship of the patient’s leg end point, the patient motion intention is obtained and used in the proposed active rehabilitation training method. The simulation experiment demonstrates the correctness of mechanism leg dynamics equations through ADAMS software and MATLAB software. The calibration experiment of the joint torque sensors’ combining limit range filter with an average value filter provides the hardware support for active rehabilitation training. The consecutive variation of the torque sensors from just the mechanism leg weight, as well as both the mechanism leg and the patient leg weights, obtains the feasibility of lower limb motion intention acquisition.

## 1. Introduction 

Cerebral vascular disease, hemiplegic, and paraplegia may cause limb motor dysfunction. For patients with limb dysfunction, the quality of life depends on the level of limb damage. Based on nerve rehabilitation theory, patients can recover through specialized rehabilitation training [1,2,3]. The lower limb rehabilitation robot is an application of robotic technology for people with lower limb disabilities [4]. In recent years, research on the lower limb rehabilitation robots has become an active topic [5,6]. Several kinds of lower limb rehabilitation robots have been developed [7]. These can be divided into the single degree-of-freedom gait trainers [8], wearable gait trainers [9,10], suspended gait trainers [11,12,13,14], and sitting/lying gait trainers [15,16]. Switzerland has developed a suspended gait trainer, Lokomat, whose left and right mechanism legs can assist patients to simulate the walking gait of normal people and restore the control ability of the nervous system to walk [17,18]. M. Bouri et al. developed a new rehabilitation robot, Lambda. Based on two translational articulations and one rotational for ankle mobilization, the patient’s hip, knee, and ankle can conveniently be mobilized in order to carry out rehabilitation, fitness, or high-level sport training [19]. Carleton University made a virtual gait rehabilitation robot (ViGRR) for bed-ridden stroke patients. It can provide average gait motion training as well as other targeted exercises, such as leg press, stair stepping, and motivational gaming [20]. Yildiz University of Science and Technology in Turkey made a sitting/lying gait trainer, Physiotherabot, helping patients to do passive training and active training [21]. A new applicable and effective sitting/lying lower limb rehabilitation robot (the LLR-Ro) is proposed in this paper.

The outstanding feature of the intelligent rehabilitation robots is that they can help the patient complete active rehabilitation training [22,23,24,25]. Active rehabilitation training is a high-level rehabilitation training method. It can improve a patient’s training initiative and accelerate the rehabilitation process, realizing patient-led training to replace traditional robot-led training. The most important part of active rehabilitation training is the acquisition of patient lower limb motion intention. There are many excellent achievements to recognize the limb motion intention. *Zhang* et al. developed a sitting/lying lower limb rehabilitation robot, named iLeg [26]. It employs the surface electromyography (EMG) signals from muscle groups to obtain the Cartesian torque/force. Based on the Cartesian torque/force, iLeg can detect the patient’s motion intention and assist the patient to achieve active rehabilitation training. Leonard et al. proposed a novel EMG-driven hand exoskeleton for stroke patients’ bilateral rehabilitation through grasping motion [27]. Yepes et al. used the optimal frame of the EMG signal to obtain the motion intention of knee joints, and adaptively derived the necessary moment to follow the motion of the knee joint based on the patient’s random motion [28]. Khoshdel et al. collected electromyogram signals near the four muscles of the lateral femoral muscle, rectus femoris, medial femoral muscle, and biceps femoris of the lower extremity through multiple channels to identify the motion and state of the knee joint [29]. However, the EMG-driven robot has its shortcomings. The EMG signal strength is changed with the patient’s leg rehabilitation and it is difficult to build the relationship between EMG signals and patient leg motion intention to realize continuing control. Present human motion intention detection is mainly designed based on biomechanical signals [30,31]. Based on the Inertial Measurement Units, Wittmann et al. proposed an arm tracking method which is used in the home environment [32]. Hwang et al. installed a number of pressure sensors on the contact surface between the three-degrees-of-freedom standing lower limb rehabilitation robot and human lower limbs, and collected human–computer interaction force information as a quantitative active motion intention [33]. A tactile control method based on plantar force signals was proposed by Berlin University of Technology, Germany, to meet the training needs of patients with arbitrary trajectories in the daily activities of lower limbs [34]. Most researchers could detect the patient motion intention based on relationships between the patient leg end forces and patient leg joint torques. However, the joint torques of the patient leg are difficult to obtain and most of them are estimated for use. This paper proposes the robot, the LLR-Ro, whose torque sensors are installed on the joint axis. Based on the dynamics modeling of human–machine coordination and the impact on the mechanism leg torques from the patient leg end force, the patient motion intention can be obtained through the variation of torque sensors installed on the mechanism leg.

## 2. Materials and Methods

### 2.1. Mechanism and Hardware Control System Design of the LLR-Ro

**Figure 1:**
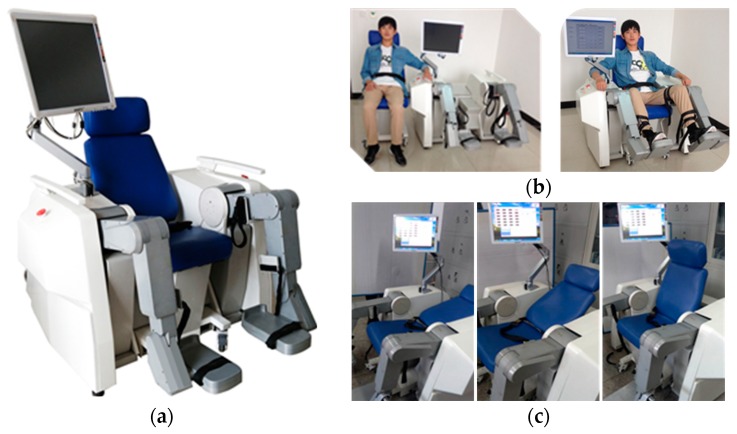
The prototype of the LLR-Ro: (**a**) The prototype of the LLR-Ro; (**b**) The movable seat separated from and grouped into the LLR-Ro; (**c**) The back angle of the movable seat altered from 110° to 170°.

For most of the sitting/lying gait trainers, their structures consist the left mechanism leg model, right mechanism leg model, chair, and so on. The structure of this trainer is just like a ring, which is difficult for patients to sit or lie on the robot. So the sitting/lying gait trainers need an auxiliary device to transfer the patient to the robots. It will cause the originally narrow rehabilitation space to become more crowded in rehabilitation institutions or hospitals. Based on the modular principle, the LLR-Ro is composed of the movable seat, the left mechanism leg model, the right mechanism leg model, the control box, and the touch screen monitor. The LLR-Ro was designed as shown in the Figure 1. There are four universal wheels under the base of the movable seat, so the movable seat can separate from the LLR-Ro to transfer the patient on the robot easily without the help of another auxiliary device. The innovative design of the movable seat can also help the patient realize sitting or lying on it fairly easily.

**Figure 2:**
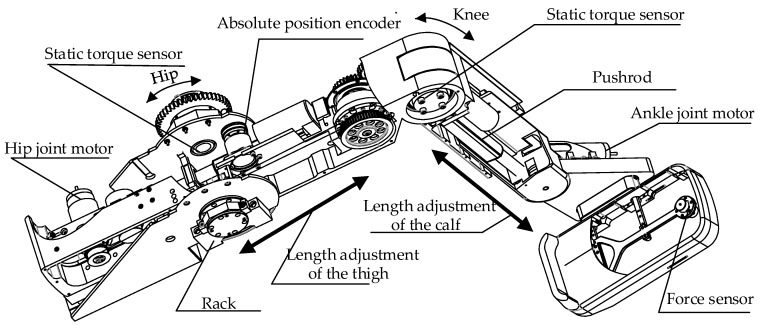
Design of the mechanism leg.

The most important part of the LLR-Ro is the mechanism leg as shown in Figure 2. The left mechanism leg module and the right mechanism leg module are bilaterally symmetrical. Each module has a mechanism leg, which has the hip, knee, and ankle joint in the human body sagittal. Based on Man–machine Engineering and the innovative design for the mechanism, the length of the mechanism leg can be automatically adjusted to fit patients with different heights through the motor-driven pushrod. The mechanism leg contains sensors to estimate the torque and force produced by patients. It also contains sensors to measure joint rotation and motors to drive the three joints. Both the hip joint and knee joint adapt a mechanical structure where the torque sensors are installed on the joint axis. Though it increases the difficulty of the mechanism leg design, it avoids the transmission errors of the joint torques.

**Figure 3:**
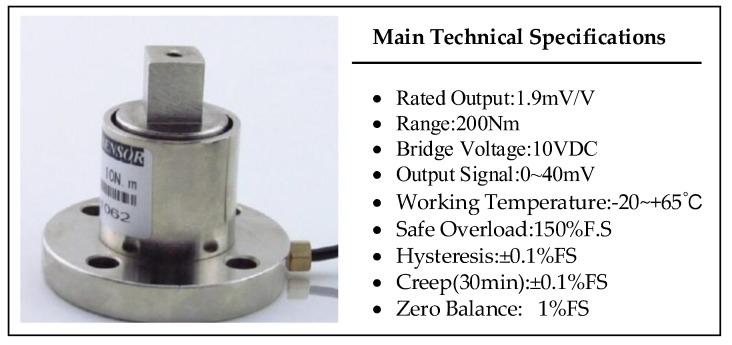
The profile and detailed parameters of the torque sensor.

The torque sensors for the hip joint and knee joint of the mechanism leg were manufactured by Shijiazhuang Baisen Instruments and Technology Co., Ltd. in China. The profile of the sensor and the sensor’s detailed parameters are shown in Figure 3, including the rated output, range, bridge voltage, and output signal.

**Figure 4:**
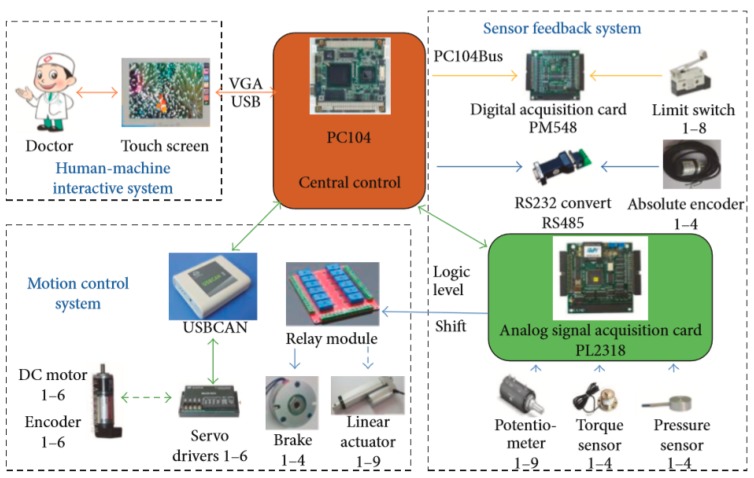
Design of the hardware control system.

Based on the functions of the LLR-Ro, the hardware control system contains the central control module, the human–machine interactive system, the sensor feedback system, and the motion control system, as shown in Figure 4. The central control module mainly runs the control software and receives the operational order from the human–machine interactive system. The human–machine interactive system displays the control software interface and feeds back the training conditions. The motion control system receives the motion control commands from the central control module, realizes the motor closed-loop control, and feeds back the joint real motion condition to the central control module. The sensor feedback system acquires the sensor information and achieves the sensor state.

### 2.2. Patient Lower Limb Motion Intention Acquisition

**Figure 5:**
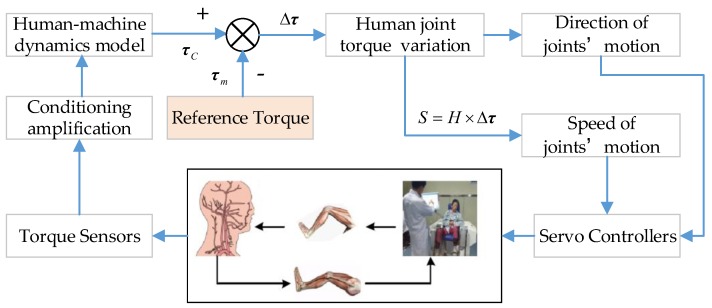
The motion intention acquisition flow diagram of the patient’s lower limb.

Based on the innovative design of the robot mechanism leg, the joint angular torques, angular positions, and angular velocities of the LLR-Ro can be measured in real time. Through the dynamics relationship between the patient leg and robot mechanism leg, and the variation of the mechanism leg joint torques, the robot can conclude the patient motion intention and help the patient realize active rehabilitation training. The flow chart of patient lower limb motion intention acquisition is shown in Figure 5. When the patient’s foot is placed on the mechanism leg without active force, the value $\tau_{m}$ is collected by the joint torque sensors. Then, the patient can begin active rehabilitation training, and the patient starts to exert force on the mechanism leg. At this time, the torques of each joint are recorded, which is called the actual torque $\tau_{C}$. By comparing $\tau_{C}$ and $\tau_{m}$, the patient’s movement intention is determined by the rehabilitation robot, and then the speed of motor movement is controlled. Considering the uncertainty of the patient motion intention and the unsteadiness of the patient’s illness, the patient’s safety is the most important factor to be considered. The variation of the torque sensor is processed through the recursion median filtering. The sampling frequency is set as 200 Hz. The sampling period is set as five samples per period. The deviation value Δτ is calculated through the average difference between data in the current moment and data in the previous moment. The control speed is obtained through the proportional control function S=H×Δτ. The proportionality coefficient *H* is set large at the start of active training or when the patient’s lower limb has low strength. It can be adjusted by the therapist to assist the patient as needed. The optimal coefficient *H* should also be determined through the clinical trial to meet patients with different levels of rehabilitation.

**Figure 6:**
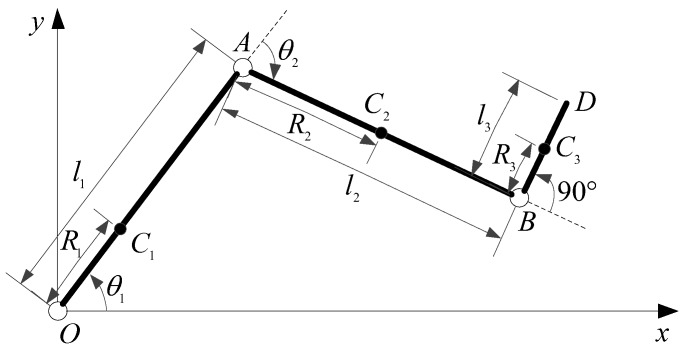
The linkage model of the mechanism leg.

The mechanism legs were simplified into a linkage model, as shown in Figure 6. O, A, and B represent the hip joint, knee joint, and ankle joint of mechanism leg, respectively. The hip joint angle and knee joint angle are expressed through $\theta_{1}$ and $\theta_{2}$, while the ankle joint angle is a constant equaling 90°. $l_{i}(i=1,2,3)$ represents the length of each segment, $C_{i}(i=1,2,3)$ represents the rod centroid, and $R_{i}(i=1,2,3)$ represents the length from centroid to joint axis.

We obtained the coordinate position of point D:

(1) $$\left\{ \begin{array}{l} x_{D}=l_{1}\cos\theta_{1}+l_{2}\cos(\theta_{1}+\theta_{2})-l_{3}\sin(\theta_{1}+\theta_{2})\\ y_{D}=l_{1}\sin\theta_{1}+l_{2}\sin(\theta_{1}+\theta_{2})+l_{3}\cos(\theta_{1}+\theta_{2}) \end{array}\right..$$

The angular position and angular velocities of each rod centroid were calculated:

(2) $$\{\begin{array}{l} x_{C1}=R_{1}\cos\theta_{1}\\ y_{C1}=R_{1}\sin\theta_{1}\\ x_{C2}=l_{1}\cos\theta_{1}+R_{2}\cos(\theta_{1}+\theta_{2})\\ y_{C2}=l_{1}\sin\theta_{1}+R_{2}\sin(\theta_{1}+\theta_{2})\\ x_{C3}=l_{1}\cos\theta_{1}+l_{2}\cos(\theta_{1}+\theta_{2})-R_{3}\sin(\theta_{1}+\theta_{2})\\ y_{C3}=l_{1}\sin\theta_{1}+l_{2}\sin(\theta_{1}+\theta_{2})+R_{3}\cos(\theta_{1}+\theta_{2})\\ {\dot{x}}_{C1}=-R_{1}{\dot{\theta }}_{1}\sin\theta_{1}\\ {\dot{y}}_{C1}=R_{1}{\dot{\theta }}_{1}\cos\theta_{1}\\ {\dot{x}}_{C2}=-{\dot{\theta }}_{1}l_{1}\sin\theta_{1}-({\dot{\theta }}_{1}+{\dot{\theta }}_{2})R_{2}\sin(\theta_{1}+\theta_{2})\\ {\dot{y}}_{C2}={\dot{\theta }}_{1}l_{1}\cos\theta_{1}+({\dot{\theta }}_{1}+{\dot{\theta }}_{2})R_{2}\cos(\theta_{1}+\theta_{2})\\ {\dot{x}}_{C3}=-{\dot{\theta }}_{1}l_{1}\sin\theta_{1}-({\dot{\theta }}_{1}+{\dot{\theta }}_{2})l_{2}\sin(\theta_{1}+\theta_{2})-({\dot{\theta }}_{1}+{\dot{\theta }}_{2})R_{3}\cos(\theta_{1}+\theta_{2})\\ {\dot{y}}_{C3}={\dot{\theta }}_{1}l_{1}\cos\theta_{1}+({\dot{\theta }}_{1}+{\dot{\theta }}_{2})l_{2}\cos(\theta_{1}+\theta_{2})-({\dot{\theta }}_{1}+{\dot{\theta }}_{2})R_{3}\sin(\theta_{1}+\theta_{2}) \end{array}.$$

Then the total kinetic energy of the mechanism leg $E_{k}$ was:

(3) $$E_{k}=E_{k1}+E_{k2}+E_{k3}=\left\{\begin{array}{l} \frac{1}{2}{\dot{\theta }}_{1}{}^{2}\left\{\begin{array}{l} m_{1}R_{1}{}^{2}+m_{2}(l_{1}{}^{2}+R_{2}{}^{2})+m_{3}(l_{1}{}^{2}+l_{2}{}^{2}+R_{3}{}^{2})+(I_{1}+I_{2}+I_{3})+\\ 2(m_{2}R_{2}l_{1}+m_{3}l_{1}l_{2})\left[\sin\theta_{1}\sin(\theta_{1}+\theta_{2})+\cos\theta_{1}\cos(\theta_{1}+\theta_{2})\right]+\\ m_{3}l_{1}R_{3}\left[\sin\theta_{1}\cos(\theta_{1}+\theta_{2})-\cos\theta_{1}\sin(\theta_{1}+\theta_{2})\right] \end{array}\right\}+\\ \frac{1}{2}{\dot{\theta }}_{2}{}^{2}\left[m_{2}R_{2}{}^{2}+m_{3}(l_{2}{}^{2}+R_{3}{}^{2})+(I_{2}+I_{3})\right]+\\ {\dot{\theta }}_{1}{\dot{\theta }}_{2}\left\{\begin{array}{l} m_{2}R_{2}{}^{2}+m_{3}(l_{2}{}^{2}+R_{3}{}^{2})+(I_{2}+I_{3})+\\ (m_{2}R_{2}l_{1}+m_{3}l_{1}l_{2})\left[\sin\theta_{1}\sin(\theta_{1}+\theta_{2})+\cos\theta_{1}\cos(\theta_{1}+\theta_{2})\right]+\\ m_{3}l_{1}R_{3}\left[\sin\theta_{1}\cos(\theta_{1}+\theta_{2})-\cos\theta_{1}\sin(\theta_{1}+\theta_{2})\right] \end{array}\right\} \end{array}\right\},$$

where $E_{ki}\left(i=1,2,3\right)$ represents the kinetic energy of the rod i, $m_{i}\left(i=1,2,3\right)$ represents the weight of the rod i and $I_{i}$ represents the rotational inertia of the rod i.

The total potential energy of the mechanism leg $E_{p}$ was:

(4) $$E_{P}=E_{P1}+E_{P2}+E_{P3}=\left[\begin{array}{l} (m_{1}gR_{1}+m_{2}gl_{1}+m_{3}gl_{1})\sin\theta_{1}+\\ \left(m_{2}gR_{2}+m_{3}gl_{2})\sin(\theta_{1}+\theta_{2})+m_{3}gR_{3}\cos(\theta_{1}+\theta_{2}\right) \end{array}\right],$$

where $E_{pi}\left(i=1,2,3\right)$ represents the potential energy of the rod i.

Lagrange function is defined as the difference between the total kinetic energy (K) and the total potential energy (P) of the mechanical system [35]:

(5) L=K−P.

By using Lagrange function, the system dynamics equation (the second Lagrange equation) [35] was:

(6) $$\tau =\frac{d}{dt}\frac{\partial L}{\partial \dot{\theta }}-\frac{\partial L}{\partial \theta },$$

where θ represents the generalized coordinates of the kinetic energy and potential energy system, $\dot{\theta }$ represents the generalized velocity of the system, and τ represents the driving torque vector.

As the potential energy $E_{P}$ did not contain $\dot{q}$ obviously, the dynamics equation was transformed into [35]:

(7) $$\tau =\frac{d}{dt}\frac{\partial E_{k}}{\partial \dot{q}}-\frac{\partial E_{k}}{\partial q}+\frac{\partial E_{p}}{\partial q}.$$

Each joint torque was calculated:

(8) $$\tau_{1}=\frac{d}{dt}\frac{\partial E_{k}}{\partial {\dot{\theta }}_{1}}-\frac{\partial E_{k}}{\partial \theta_{1}}+\frac{\partial E_{p}}{\partial \theta_{1}}=\left\{\begin{array}{l} {\ddot{\theta }}_{1}\left\{\begin{array}{l} m_{1}R_{1}{}^{2}+m_{2}(l_{1}{}^{2}+R_{2}{}^{2})+m_{3}(l_{1}{}^{2}+l_{2}{}^{2}+R_{3}{}^{2})+(I_{1}+I_{2}+I_{3})+\\ 2(m_{2}R_{2}l_{1}+m_{3}l_{1}l_{2})\left[\sin\theta_{1}\sin(\theta_{1}+\theta_{2})+\cos\theta_{1}\cos(\theta_{1}+\theta_{2})\right]+\\ m_{3}l_{1}R_{3}\left[\sin\theta_{1}\cos(\theta_{1}+\theta_{2})-\cos\theta_{1}\sin(\theta_{1}+\theta_{2})\right] \end{array}\right\}+\\ {\ddot{\theta }}_{2}\left\{\begin{array}{l} m_{2}R_{2}{}^{2}+m_{3}(l_{2}{}^{2}+R_{3}{}^{2})+(I_{2}+I_{3})+\\ (m_{2}R_{2}l_{1}+m_{3}l_{1}l_{2})\left[\sin\theta_{1}\sin(\theta_{1}+\theta_{2})+\cos\theta_{1}\cos(\theta_{1}+\theta_{2})\right]+\\ m_{3}l_{1}R_{3}\left[\sin\theta_{1}\cos(\theta_{1}+\theta_{2})-\cos\theta_{1}\sin(\theta_{1}+\theta_{2})\right] \end{array}\right\}+\\ {\dot{\theta }}_{1}{\dot{\theta }}_{2}\left\{\begin{array}{l} 2(m_{2}R_{2}l_{1}+m_{3}l_{1}l_{2})\left[\sin\theta_{1}\cos(\theta_{1}+\theta_{2})-\cos\theta_{1}\sin(\theta_{1}+\theta_{2})\right]-\\ m_{3}l_{1}R_{3}\left[\sin\theta_{1}\sin(\theta_{1}+\theta_{2})+\cos\theta_{1}\cos(\theta_{1}+\theta_{2})\right] \end{array}\right\}+\\ {\dot{\theta }}_{2}{}^{2}\left\{\begin{array}{l} (m_{2}R_{2}l_{1}+m_{3}l_{1}l_{2})\left[\sin\theta_{1}\cos(\theta_{1}+\theta_{2})-\cos\theta_{1}\sin(\theta_{1}+\theta_{2})\right]-\\ m_{3}l_{1}R_{3}\left[\sin\theta_{1}\sin(\theta_{1}+\theta_{2})+\cos\theta_{1}\cos(\theta_{1}+\theta_{2})\right] \end{array}\right\}+\\ (m_{1}gR_{1}+m_{2}gl_{1}+m_{3}gl_{1})\cos\theta_{1}+\\ \left(m_{2}gR_{2}+m_{3}gl_{2})\cos(\theta_{1}+\theta_{2})-m_{3}gR_{3}\sin(\theta_{1}+\theta_{2}\right) \end{array}\right\},$$

(9) $$\tau_{2}=\frac{d}{dt}\frac{\partial E_{k}}{\partial {\dot{\theta }}_{2}}-\frac{\partial E_{k}}{\partial \theta_{2}}+\frac{\partial E_{p}}{\partial \theta_{2}}=\left\{\begin{array}{l} {\ddot{\theta }}_{2}\left[m_{2}R_{2}{}^{2}+m_{3}(l_{2}{}^{2}+R_{3}{}^{2})+(I_{2}+I_{3})\right]-\\ \frac{1}{2}{\dot{\theta }}_{1}{}^{2}\left\{\begin{array}{l} 2(m_{2}R_{2}l_{1}+m_{3}l_{1}l_{2})\left[\sin\theta_{1}\cos(\theta_{1}+\theta_{2})-\cos\theta_{1}\sin(\theta_{1}+\theta_{2})\right]-\\ m_{3}l_{1}R_{3}\left[\sin\theta_{1}\sin(\theta_{1}+\theta_{2})+\cos\theta_{1}\cos(\theta_{1}+\theta_{2})\right] \end{array}\right\}+\\ {\ddot{\theta }}_{1}\left\{\begin{array}{l} m_{2}R_{2}{}^{2}+m_{3}(l_{2}{}^{2}+R_{3}{}^{2})+(I_{2}+I_{3})+\\ (m_{2}R_{2}l_{1}+m_{3}l_{1}l_{2})\left[\sin\theta_{1}\sin(\theta_{1}+\theta_{2})+\cos\theta_{1}\cos(\theta_{1}+\theta_{2})\right]+\\ m_{3}l_{1}R_{3}\left[\sin\theta_{1}\cos(\theta_{1}+\theta_{2})-\cos\theta_{1}\sin(\theta_{1}+\theta_{2})\right] \end{array}\right\}+\\ \left(m_{2}gR_{2}+m_{3}gl_{2})\cos(\theta_{1}+\theta_{2})-m_{3}gR_{3}\sin(\theta_{1}+\theta_{2}\right) \end{array}\right\}.$$

The dynamics equation was simplified as follows [35]:

(10) $$H(\theta )\ddot{\theta }+C(\theta ,\dot{\theta })\dot{\theta }+G(\theta )=\tau ,$$

where, H(θ) represents the inertia matrix, $C(\theta ,\dot{\theta })$ represents the centrifugal force and the Coriolis force matrix, and G(θ) represents the gravity matrix.

The contact force between the lower limb of the patient and the end of the mechanism leg changed in real time with the movement of the mechanism leg. When we just considered the effect on the joint torque of the mechanism leg from patient leg weight, the joint torque $\tau_{p}$ generated by the endpoint force was obtained based on the force Jacobin formula [35]:

(11) $$\tau_{p}=J^{T}(\theta )F_{0},$$

where $J^{T}(\theta )$ represents the force Jacobin matrix of the mechanism legs and $F_{0}$ represents the contact force between the patient’s leg and the mechanism leg while the patient does not exert active force.

Combined with the mechanism leg’s kinematics, the expression $J^{T}(\theta )$ was obtained:

(12) $$J^{T}(\theta )=\left[\begin{array}{cc} -l_{1}\sin\theta_{1}-l_{2}\sin(\theta_{1}+\theta_{2}) & l_{1}\cos\theta_{1}+l_{2}\cos(\theta_{1}+\theta_{2})\\ -l_{2}\sin(\theta_{1}+\theta_{2}) & l_{2}\cos(\theta_{1}+\theta_{2}) \end{array}\right].$$

Combined with the robot dynamics equation, the dynamics modeling of human–machine coordination was obtained when the patient’s leg was put on the rehabilitation robot:

(13) $$H(\theta )\ddot{\theta }+C(\theta ,\dot{\theta })\dot{\theta }+G(\theta )=\Delta \tau =\tau -J^{T}(\theta )F_{0}.$$

## 3. Results

### 3.1. Verification of the Mechanism Leg Dynamics Equations

**Figure 7:**
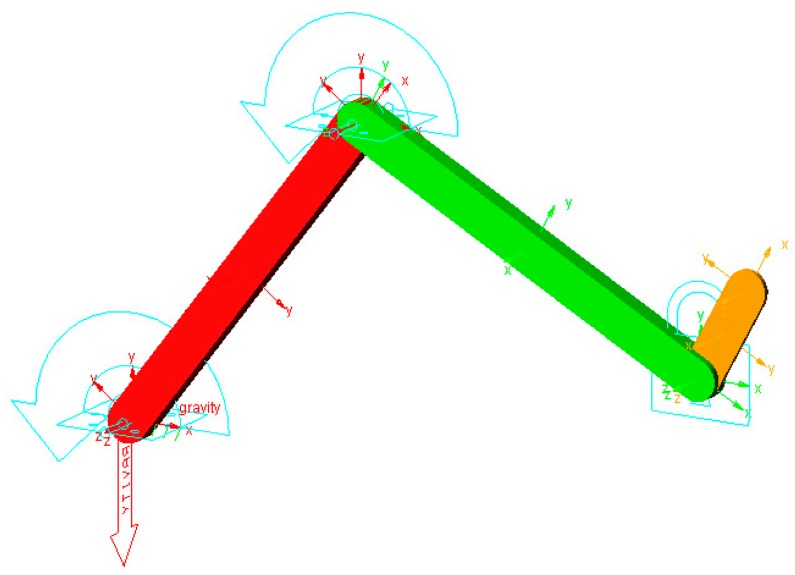
Simulation model developed through ADAMS.

The dynamics equations of the LLR-Ro were so complicated and the solution procedure was tedious. It is very necessary to prove the correctness of the derivative results. The verification of the dynamics equation was conducted through ADAMS software and MATLAB software. ADAMS software, designed by American MSC Company, was used for the multi-body dynamics simulation. Figure 7 shows the simulation model developed through ADAMS.

The model is a three linkage manipulator. It has three joints moving in xy-plane. The first joint is the hip joint and the second is the knee joint. The third joint is the ankle joint and it is locked. The detailed parameters of the three linkage manipulator are given as below in Table 1.

The simulation time was t=8 s, and the driving function of the hip joint is given as below:

(14) $$\left\{ \begin{array}{l} \theta_{11}(t)=10+4.935t^{2}-0.603t^{3}+0.011t^{4}\left(0\leq t\leq 4\right)\\ \theta_{12}(t)=53.216+13.384t_{1}-1.234t_{1}{}^{2}-0.425t_{1}{}^{3}+0.066t_{1}{}^{4}\left(t_{1}=t-4;4\leq t\leq 8\right) \end{array}\right.,$$

where $\theta_{11}$ and $\theta_{12}$ are the hip joint angular position at the times 0≤t≤4 and 4≤t≤8, respectively.

The driving function of the knee joint is given as below:

(15) $$\left\{ \begin{array}{l} \theta_{21}(t)=-11.390t^{2}+1.967t^{3}-0.098t^{4}\left(0\leq t\leq 4\right)\\ \theta_{22}(t)=-81.350-21.691t_{1}+2.848t_{1}{}^{2}+0.407t_{1}{}^{3}-0.081t_{1}{}^{4}\left(t_{1}=t-4;4\leq t\leq 8\right) \end{array}\right.,$$

where $\theta_{21}$ and $\theta_{22}$ are the knee joint angular position at the times 0≤t≤4 and 4≤t≤8, respectively.

Then we obtained the hip joint and knee joint actual driving torque through ADAMS, as shown in Figure 7. Meanwhile, we also achieved the theoretical driving torques based on the dynamics Equations (8) and (9). According to the driving function of joints, the velocity and accelerations of the hip and knee joints were obtained as below:

(16) $$\{\begin{array}{l} {\dot{\theta }}_{11}(t)=9.870t-1.809t^{2}+0.044t^{3}(0\leq t\leq 4)\\ {\ddot{\theta }}_{11}(t)=9.870-3.618t+0.132t^{2}{\left(0\leq t\leq 4\right)}_{}\\ {\dot{\theta }}_{12}(t)=13.384-2.468t_{1}-1.275t_{1}{}^{2}+0.264t_{1}{}^{3}{\left(t_{1}=t-4,4\leq t\leq 8\right)}_{}\\ {\ddot{\theta }}_{12}(t)=-2.468-2.550t_{1}+0.792t_{1}{}^{2}{\left(t_{1}=t-4,4\leq t\leq 8\right)}_{}\\ {\dot{\theta }}_{21}(t)=-22.780t+5.901t^{2}-0.392t^{3}{\left(0\leq t\leq 4\right)}_{}\\ {\ddot{\theta }}_{21}(t)=-22.780+11.802t-1.176t^{2}(0\leq t\leq 4)\\ {\dot{\theta }}_{22}(t)=-21.691+5.696t_{1}+1.221t_{1}{}^{2}-0.324t_{1}{}^{3}(t_{1}=t-4,4\leq t\leq 8)\\ {\ddot{\theta }}_{22}(t)=5.696+2.442t_{1}-0.972t_{1}{}^{2}(t_{1}=t-4,4\leq t\leq 8) \end{array}.$$

**Figure 8:**
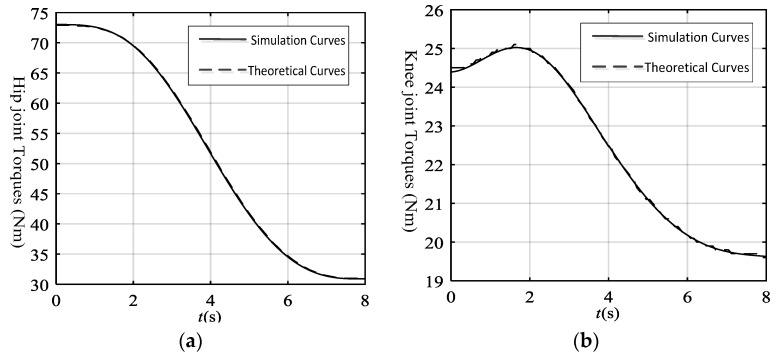
The simulation curves and theoretical curves of the mechanism leg joints: (**a**) The simulation curves and theoretical curves of the hip joint; (**b**) The simulation curves and theoretical curves of the knee joint.

The theoretical driving torques were also obtained through MATLAB, as shown in Figure 8. Based on the contrast curves, the theoretical curves basically fit with the actual curves. So we could conclude that the dynamics equations of the LLR-Ro are correct.

### 3.2. Calibration Experiment of the Joint Static Torque Sensors

**Figure 9:**
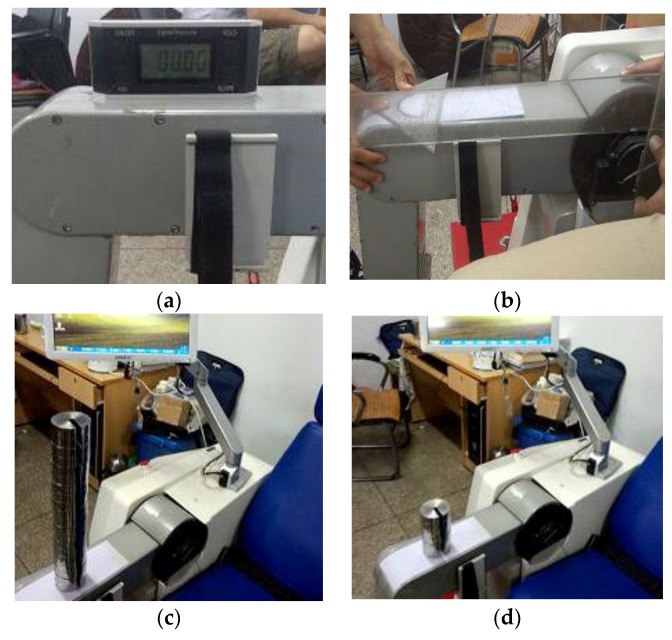
The calibration experiment of torque sensors: (**a**) The thigh of the mechanism leg is set at the horizontal position; (**b**) One point is marked from the hip joint axis 585 mm; (**c**) The analytical weights (each weight is 2.5 kg) are put on the marked point one by one until the weight equals 17.5 kg; (**d**) The weights start to be unloaded one by one until it equals 0 kg. The steps above are repeated three times, and the voltage values are processed through the combing limit range filter and average value filter.

In active training, the torque sensors are important for the whole control. The calibration experiment of torque sensors was conducted. The voltage values of the torque sensors were obtained through the analogue acquisition PL2318. The voltage values were processed through the combing limit range filter with an average value filter. The merit of this filtering method is that it can overcome accidental jamming and the curve of the voltage value is smooth. The calibration of the hip joint torque sensor was similar to the calibration of the knee joint torque sensor. The detailed calibration process of the hip joint torque is introduced in Figure 9.

**Figure 10:**
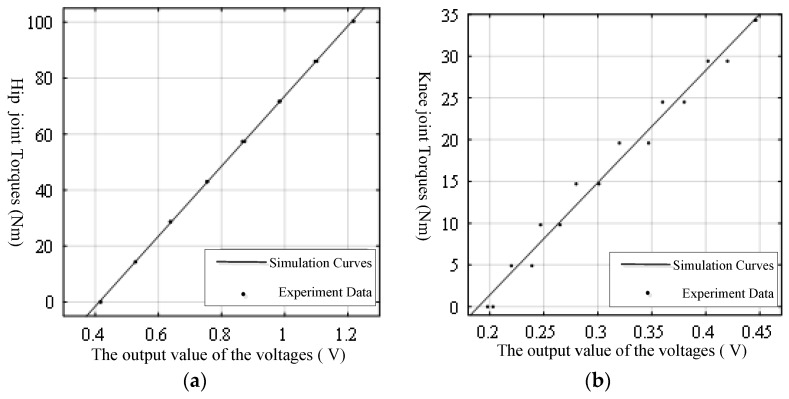
The calibration curves of the joint torque sensors: (**a**) The calibration curves of the hip joint torque sensors; (**b**) The calibration curves of the knee joint torque sensors.

Through data processing, the calibration curves of the hip and knee joint torque sensors were obtained, as shown in Figure 10. The curves can be described through the below expression:

(17) $$\left\{ \begin{array}{l} M_{1}=125.3V_{1}\\ M_{2}=134.5V_{2} \end{array}\right.,$$

where $M_{1}$ and $M_{2}$ represent the hip joint torque and the knee joint torque, and their units are Nm, and $V_{1}$ and $V_{2}$ represent the voltage value of hip joint torque and knee joint torque, and their units are *V*.

### 3.3. Verification Experiment of the Motion Intension Acquisition Based on Biomechanics

**Figure 11:**
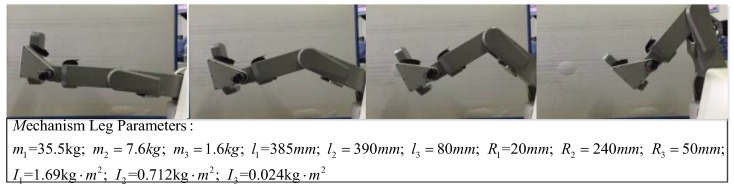
The experiment to obtain the joint torques from the mechanism leg weight.

**Figure 12:**
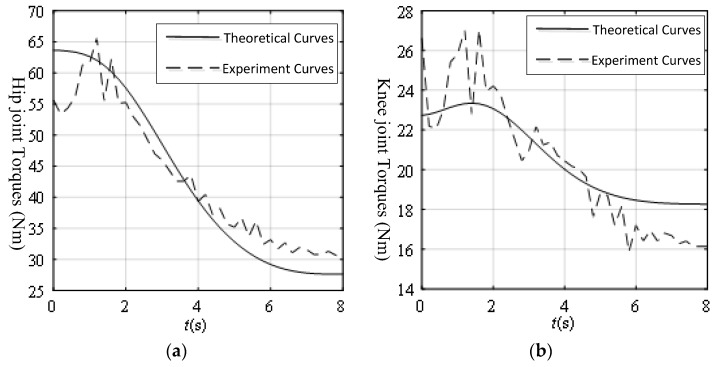
The calibration curves of the joint torque sensors: (**a**) Hip torque just from the mechanism leg weight; (**b**) Knee torque just from the mechanism leg weight.

Based on the calibration experiment of the joint torque sensors, there existed errors between the actual torques and the theoretical torques. It was necessary for the joint torques to set a given threshold values. The given threshold values were obtained through the experiment, as shown in Figure 11. Then theoretical torques and the actual torques just from the mechanism leg weight were also obtained, as shown in Figure 12. The experiment curves were similar to the theoretical curves. However, the errors were very large at the start of the experiment, because of the heavy mechanism leg and the mechanism assembly error. The maximum errors of the hip joint and the knee joint from the mechanism leg weight were 9.82 Nm and 3.89 Nm, respectively. So the given threshold values of the hip joint torque and the knee joint torque were set to 10 Nm and 5 Nm, respectively.

**Figure 13:**
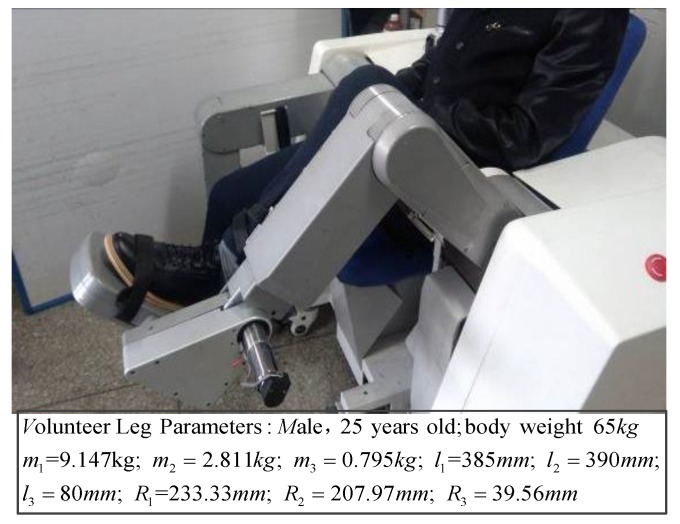
The verification experiment of active training without patient active force.

One volunteer participated in the experiment and his leg was put on the mechanism leg, as shown in Figure 13.

**Figure 14:**
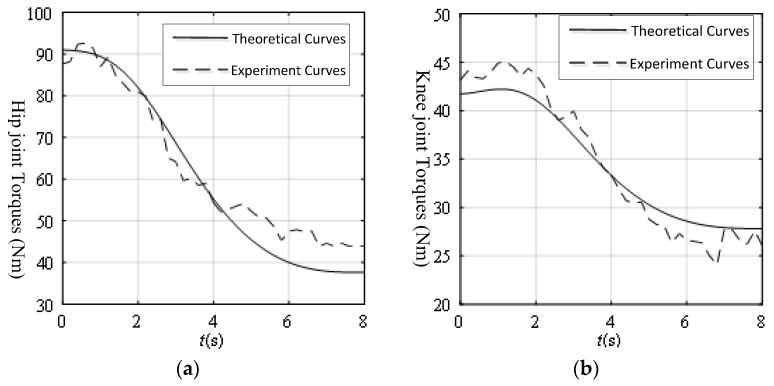
Joint torques from the mechanism leg and patient leg weights: (**a**) Hip torque from the mechanism leg and patient leg weights; (**b**) Knee torque from the mechanism leg and patient leg weights.

Figure 14 shows the theoretical and experiment joint torque curves from the mechanism leg and volunteer leg weight. The maximum error of the hip joint without active force was 9.08 Nm and the maximum error of the knee joint without active force was 3.91 Nm.

## 4. Discussion

From the calibration curves of the joint torque sensors in Figure 10, the actual test points were closely distributed around the fitting curves, reflecting that the torque sensor had better linear characteristics. However, because of the complex structure of the components around the torque sensor, and the processing accuracy, installation error, and the wear and amplification error of the conditioning board, there was a deviation between the function relationship between the voltage value of the torque sensor in the real state and the ideal state.

At the start, the errors in Figure 14 were smaller than the errors in Figure 12 because the volunteer’s leg weights were just like a pre-tightening force, making the transmission error on the mechanism leg smaller. It was demonstrated that the proposed threshold values of the joint torques were satisfied for future experiments. All the errors in Figure 14 were in the scope of the threshold values. From Figure 12 and Figure 14, although the volunteer’s leg weight was much lighter than the mechanism leg weight, the variations of the joint torques were prominent. If the patient exerted his active force on the mechanism leg, the variations of joint torques could be much larger. Based on the variations of the joint torques, the principle of detecting the volunteer motion intention is clear and feasible.

Compared with other methods, like EMG, for obtaining patient motion intention, this method may be more suitable for continuous active rehabilitation training control. This is because the EMG signals would be reduced with the patient limb becoming stronger, while the variations of the LLR-Ro torque sensors would be increased. In the future, the research team will continue conducting clinical trials to verify the excellent effect of the active rehabilitation training based on the biomechanics and differential analysis through biomechanics signals and EMG signals on the LLR-Ro.

## 5. Conclusions

This paper investigates a new applicable and effective sitting/lying multi-joint lower limb rehabilitation robot. In order to improve patient’s training initiative and accelerate the rehabilitation process, a new motion intention acquisition method based on biomechanics is proposed. The simulation experiment demonstrates the correctness of the mechanism leg dynamics equations, the calibration experiment of the joint torque sensors provides the hardware support for active rehabilitation training, and the consecutive variation of the torque sensors from just the mechanism leg weight and both the mechanism leg and patient leg weights obtains the feasibility of lower limb motion intention acquisition. In the future, new active rehabilitation training for the LLR-Ro will be proposed on the basis of the motion intention acquisition method in this paper. Meanwhile, the patients’ recovery efficiency through the future active rehabilitation training method will be verified in clinical trials.

## Figures and Tables

**Table 1:** The parameters of the three linkage manipulator.

Parameters	Thigh	Calf	Foot
Segment Length	390 mm	400 mm	100 mm
Distance from Centroid to the Joint Axis	50 mm	250 mm	50 mm
Segment Rotational Intertia	1.5 kg·m^2^	0.2 kg·m^2^	0.02 kg·m^2^
